# 

**Published:** 1881-02

**Authors:** 


### CONTENTS:

| ORIGINAL. | | Page. |
|---|---|---|
| Editoral, | | 41 |
| The Legion of Honor, | Ad. Lippe, M. D., Phila. | 44 |
| My Address at Milwaukee, | E. W. Berridge, M. D., London, | 46 |
| Fatal Errors. | Ad. Lippe, M. D., Phila. | 47 |
| Quantum Sufficit. | C. F. Nichols, M. D., Boston, | 52 |
| Differential Diagnosis of Merc. Viv. and Podophyl., | C. Carlton Smith, M. D., Phila. | 57 |
| The What Is It? | C. Pearson, M. D., Washington, | 60 |
| CLINICAL CASES: | | |
| Dropsey from Malaria, | W. H. Jenny, M. D., Iowa City, | 64 |
| Euthanasia; Caries of Jaw, | Geo. C. Gale, M. D., Quebec, | 66 |
| Cases. | E. W. Berridge, M. D., London, | 67 |
| Constipation—Silicea, | E. B. Nash, M. D., Cortland, N.Y. | 69 |
| BOOK NOTICES AND REVIEWS. | | |
| Medical Heresis, &c., by G. C. Symthe, M. D., Priestly Blakiston, | | 72 |
| Is Consumption Contagious, by Herbert C. Clapp, M. D. (Otus, Clapp & Son.) | | 78 |
| International Hahnemann Association. | | 79 |

